# Molecular cytogenetic studies characterizing a novel complex karyotype with an uncommon 5q22 deletion in childhood acute myeloid leukemia

**DOI:** 10.1186/s13039-015-0167-y

**Published:** 2015-08-07

**Authors:** Amanda Faria de Figueiredo, Roberto Rodrigues Capela de Matos, Moneeb A. K. Othman, Thomas Liehr, Elaine Sobral da Costa, Marcelo Geradin Poirot Land, Raul C. Ribeiro, Eliana Abdelhay, Maria Luiza Macedo Silva

**Affiliations:** Bone Marrow Unit, Cytogenetics Department, National Cancer Institute, Rio de Janeiro, Brazil; Post-Graduation Program in Oncology, National Cancer Institute, Rio de Janeiro, Brazil; Institute for Human Genetics, Jena University, Jena, Germany; Clinical Medicine Postgraduate Program, College of Medicine, Federal University, Rio de Janeiro, Brazil; Martagão Gesteira Institute of Pediatrics and Child Development, Federal University of Rio de Janeiro, Rio de Janeiro, Brazil; Department of Oncology, St. Jude Children’s Research Hospital, Memphis, TN USA; Bone Marrow Unit, Stem Cell Department, National Cancer Institute, Rio de Janeiro, Brazil

**Keywords:** Childhood AML, Uncommon deletion 5q22, Complex karyotype

## Abstract

Deletions in the long arm of chromosome 5 or loss of the whole chromosome are rare in childhood Acute Myeloid Leukemia (AML) patients. It is also unknown if the wide variety of breakpoints have diverging implications in the patient’s outcome. Despite -5/5q- abnormalities have usually been described as a poor prognostic feature, however, the low frequency of -5/5q- in pediatric AML patients limits a full knowledge about this cytogenetic and clinical category, which is an intriguing factor for further research and new findings. Here, we report an AML child showing an uncommon deletion in 5q associated with 2 new abnormalities involving chromosome 2 within a complex karyotype well-characterized by several molecular cytogenetic approaches. Our work stimulates upcoming studies with more detailed descriptions about 5q abnormalities to better define its role in the stratification risk of such cytogenetic subgroup in childhood AML.

## Background

Several studies in childhood acute myeloid leukemia (AML) have been showing three prognostic cytogenetic group classification: favorable cytogenetics, that includes t(8;21), t(15;17) and inv(16), high-risk cytogenetics, that includes complex karyotypes, monosomy 7, monosomy 5, del(5)(q) or abnormalities in 3q, and intermediate risk: other changes [1]. The subgroup that has -5/5q rearrangements is rare and comprises about 1−2.5 % of pediatric AML cases [2]. This finding was recently confirmed by Johnston et *al*., which described cytogenetics data of 26 pediatric patients (1.2 %) with -5/5q abnormalities in a retrospective study, including 2240 children and observed that this cytogenetic subgroup presented a very poor outcome [3]. Despite of a concise and comparative study in a large cohort of childhood AML, the low frequency of 5q- cases limit a full comprehension of the cytogenetic and clinical implications of this subgroup [1, 3, 4]. Thus, to contribute to the understanding of this rare subgroup within childhood AML, we describe the clinical, G-banding and molecular cytogenetic data of a child with AML showing an uncommon deletion 5q22 in a new complex karyotype.

## Case presentation

### Case report

A 10-year-old boy was admitted with a 4-months history of fever, generalized lymphadenopathies and weight loss. Physical examination revealed mucositis and gingival hyperplasia. The abdomen was painless with no palpable mass. Imaging examination revealed mediastinal widening. At admission, the hemoglobin was 7.2g/L, white blood cell count was 67.5x10^9^/L and platelet count was 275x10^9^/L. The peripheral blood smear showed 80 % of blast cells. Bone marrow was hypercellular with 94,6 % FAB-M1 myeloid blast cells, being 93,7 % of granulocytic progenitors (CD34^hi^; MPO^-/+(50 %)^; CD117^hi^; HLA-DR^hi^; CD13^hi^; CD36^−^; CD11b^−^; CD16^−^; CD64^−^; CD35^−^; CD14^−^; IREM-; CD71^lo^; CD105^−^; CD33^-/+(50 %)^; TdT^+^; CD7^-/+(5 %)^; CD38^+^, CD2^+^; CD15^+^). The patient was initially classified for intermediate-risk arm, and treated on AML-BFM 2012 protocol [5].

Morphological examination of the bone marrow on days 15 and 33 of induction chemotherapy revealed myeloid blast cells comprising >25 % (M3 bone marrow evaluation) and M2 (- >5 to 25 %), respectively. At the 42nd day from the beginning of the treatment, the blast cell percentage in the bone marrow was 21,6 %. The patient achieved a late complete remission (0.7 % blast cells, < 5 % - M1) only on day 79 of treatment. Due to the persistence of blast cells after the second induction (42nd day from the beginning of the treatment), the patient was stratified to high-risk arm, and was submitted to allogeneic stem cell transplantation, as recommend by the protocol [5]. This procedure was well tolerated and the patient is in continuous complete remission for eight months now and twelve months since initial diagnosis. This study was approved by the Ethics Committee of the Brazilian National Cancer Institute (CEP #088/07).

### Methods and results

**Fig. 1:**
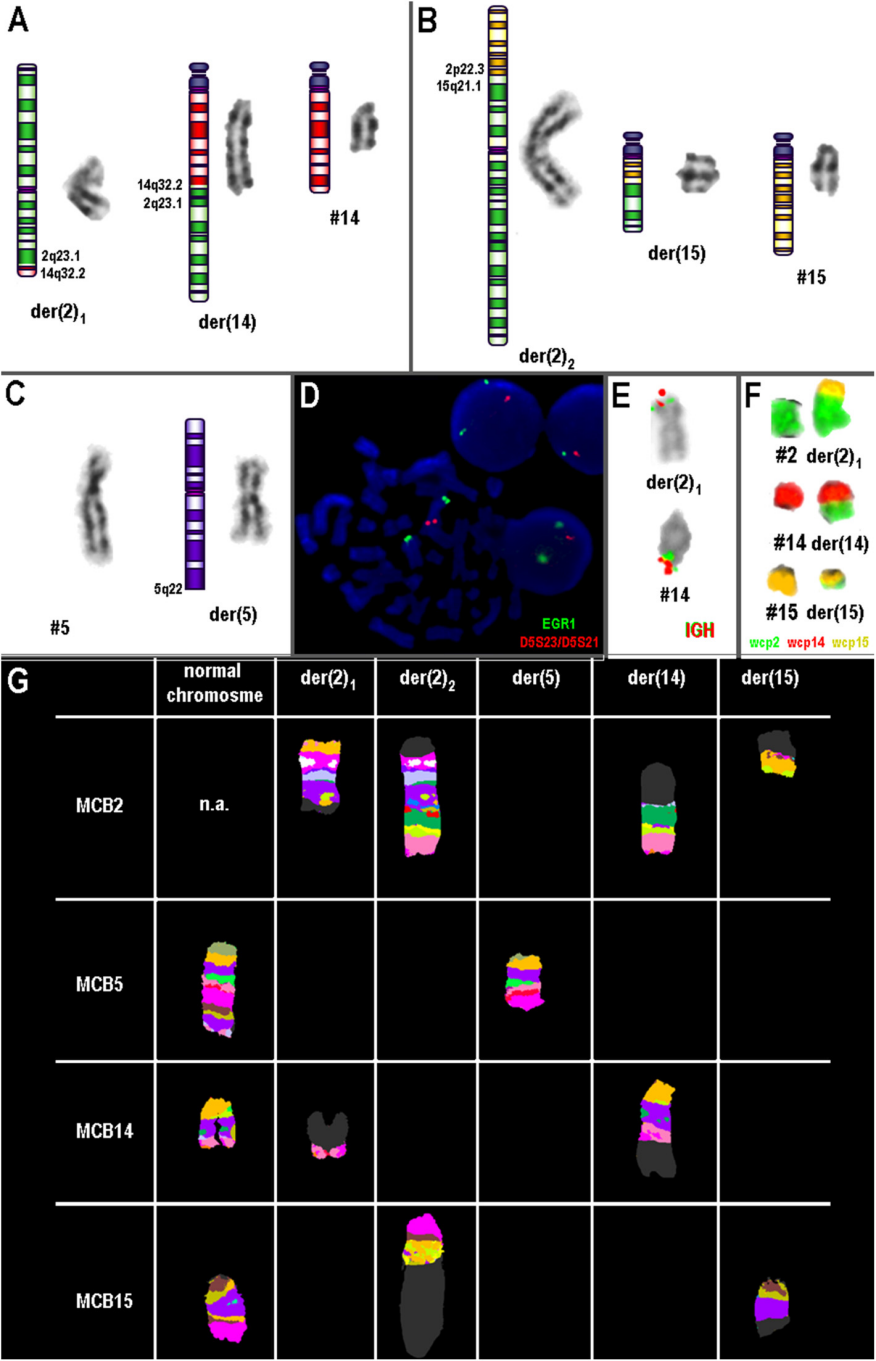
**a**, **b**, and **c** show the final partial G-banding karyotype. **a** t(2;15)(p22.3;q21.1); **b** del(5)(q22); **c** t(2;14)(q23.1;q32.2). **d** FISH with LSI 5p15/5q31 (EGR1) showing a deletion of the red signal (5q31) in a metaphase and in nuclei interphase. **e** FISH with WCP for chromosome 2 (green), 14 (red) and 15 (yellow) shows translocation between chromosomes 2, 14 and 15 (R-DAPI image). **f** FISH-results characterizing the normal and derivative chromosomes 2, 5, 14 and 15 are summarized. In the first line results of MCB for chromosome 2 are depicted and show both derivatives 2 and derivative 14 and 15. The second line shows the normal chromosome 5 and a derivative of chromosome 5. The third line shows the normal chromosome 14 and the derivative chromosomes 2 and 14. The fourth line shows the normal chromosome 15 and the derivative chromosomes 2 and 15. Here, MCB probe sets for chromosomes 2, 5, 14 and 15 were used. According to FISH and MCB results, the karyotype was re-interpreted as 46,XY, t(2;14)(q23.1;q32.2), t(2:15)(p22.3;q21.1) and del(5)(q22-qter)

G-banding studies revealed a karyotype 46,XY,der(2)?t(2;15),del(5), der(14)?add(14)(q23) in 15 of 20 metaphases analyzed (Figs. 1a, b and c). Several molecular approaches were performed to discover this complex karyotype. FISH was performed using the LSI *EGR1* SpectrumOrange/D5S23 (5q31), D5S721 SpectrumGreen (5p15) probes (Vysis) and showed a heterozygous *EGR1* deletion in 10 metaphases analyzed and interphase nuclei analysis revealed the same pattern in 150 of 200 analyzed cells (Fig. 1d). Although FISH using LSI *IGH* break apart showed no split signal (Fig. 1e), the position of IGH normal signal suggested that there was a translocation between the chromosomes 2 and 14 (Fig. 1e). Whole Chromosome Painting (WCP) probes for chromosomes 2, 14 and 15 were also applied and the results revealed a translocation between chromosomes 2 and 14 and also between the other homolog of chromosome 2 and chromosome 15, therefore the karyotype had no normal chromosome 2 (Fig. 1f). Further, Multicolor Chromosome Banding (MCB) was performed for chromosomes 2, 5, 14 and 15, as previously reported [6]. Overall, MCB characterized a complex chromosomal alteration between chromosomes 2, 14 and 15 (Fig. 1g). In order to define all the breakpoints involved in this complex translocation, mainly 5q-, it was necessary to apply several Bacterial Artificial Chromosome (BAC) probes (Table 1). The final karyotype was characterized as: 46,XY,t(2;14)(q23.1;q32.2),t(2:15)(p22.3;q21.1),del(5)(q22-qter). The karyotype was described according to the International System for Human Cytogenetic Nomenclature [7].

**Table 1:** Probes and BACs applied in the case to characterize the breakpoint in each chromosome involved

Cytoband	Position [hg19]	Probe	Results in derivative chromosomes
2p24.3	chr2: 16,014,784-16,140,647	RP11-119F22	Signal on der(15); no split signal
2q23.3	chr2: 26,967,697-27,136,688	RP11-106G13	Signal on der(15); no split signal
2p23.2	chr2: 29,415,640-29,447,593	SPEC ALK	Signal on der(15); no split signal
2p22.3	chr2:35,864,069-36,032,088	RP11-119B15	Signal on both der(2); no split signal
2q22.3	chr2:145,181,324-145,355,222	RP11-64O2	Signal split on der(2) and on der(14)
2q23.3	chr2:153,589,449-153,743,069	RP11-58K7	Signal on der(14); no split signal
5q15	chr5:93,905,245-93,906,381	RP11-461G12	Signal on der(5)
5q15	chr5:95,549,050-95,550,779	RP11-254I22	Signal on der(5)
5q15	chr5:97,013,251-97,014,385	RP11-72K17	Signal on der(5)
5q21.1	chr5:98,701,067-98,853,238	RP11-102H6	Signal on der(5)
5q21.1	chr5:100,241,673-100,434,269	RP11-109H23	Signal on der(5)
5q21.3	chr5:106,587,839-106,772,024	CTD-2337D22	Signal on der(5)
5q22.2	chr5:112,045,171-112,208,641	RP11-107C15	Signal deleted on der(5)
5q22.2	chr5:112,460,936-112,667,499	RP11-467F22	Signal deleted on der(5)
5q22.3	chr5:114,413,269-114,575,374	RP11-115D4	Signal deleted on der(5)
5q23.1	chr5:116,078,687-116,097,905	RP11-249M12	Signal deleted on der(5)
5q23.2	chr5:123,826,090-123,997,725	RP11-689H7	Signal deleted on der(5)
5q23.3	chr5:126,087,655-126,112,178	RP11-434D11	Signal deleted on der(5)
5q23.3	chr5:130,278,846-130,432,829	RP11-114H7	Signal deleted on der(5)
5q31.1	chr5:131,789,105-131,949,164	RP11-729C24	Signal deleted on der(5)
5q31.1	chr5:135,712,100-135,888,152	RP11-114H21	Signal deleted on der(5)
5q31.2	chr5:137,829,080-137,832,903	LSI EGR1	Signal deleted on der(5)
5q35.3	chr5:180,578,142-180,778,814	D5S2907	Signal deleted on der(5)
14q32.2	chr14:98,088,984-98,162,995	RP11-76E12	Signal on der(14); no split signal
14q32.2	chr14:100,070,893-100,092,077	RP11-543C4	Signal on der(2q); no split signal
14q32.33	Chr14:106,053,226- 106,518,932	LSI IGH	Signal on der(2q); no split signal
15q15.1	chr15:41,796,423-41,967,115	RP11-380D11	Signal on der(15); no split signal
15q21.1	chr15:45,625,708-45,826,511	RP11-519G16	Signal on der(15); no split signal
15q21.1	chr15:46,258,718-46,459,407	RP11-315O8	Signal on der(2), no split signal
15q21.1	chr15:48,509,039-48,663,778	RP11-154J22	Signal on der(2), no split signal
15q21.1	chr15:49,049,766-49,223,905	RP11-485O10	Signal on der(2);no split signal
15q21.1	chr15:49,896,865-50,081,728	RP11-353B9	Signal on der(2); no split signal
15q21.2	chr15:50,385,284-50,543,688	RP11-416K5	Signal on der(2); no split signal
15q21.2	chr15:50,586,357-50,763,569	RP11-802B2	Signal on der(2); no split signal
15q21.3	chr15:53,791,870-53,948,902	RP11-232J12	Signal on der(2); no split signal

### Discussion

Abnormalities of chromosome 5 are a common finding in patients with hematological malignancies with poor outcome [8]. The deletion of the long arm of chromosome 5 and monosomy of 5 has been described most frequently in Myelodysplastic Syndrome (MDS) cases. However, it has been already documented as a recurring finding in AML and it is associated with dismal outcome [9]. 5q deletion has been shown a wide variety of breakpoints and it has been reported within complex karyotypes.

Johnston and coworkers described in a wide retrospective cohort of 2240 pediatric AML patients, that 5q deletion was also associated with poor outcome, in which to have more than three cytogenetic abnormalities showed worse outcomes than those with three or fewer cytogenetic abnormalities [3]. On the other hand, it is important to remark that 7/26 patients, from the above-mentioned work were classified as harboring unbalanced rearrangements resulting in 5q loss, all of that without thoroughly refine the karyotype in the search for the presence of cryptic abnormalities, which may be particularly hard to detect in G-banding cytogenetics.

In the present work, the patient carries on the deletion of chromosome 5 within a complex karyotype and has lost a large portion from the long arm of one of the chromosomes 5. Initially, the FISH assay, with LSI probes, demonstrated the deletion of 5q31, but with FISH using several BAC probes (Table 1) and MCB approaches, we could detect the breakpoint in 5q22 which is an uncommon finding in patients harboring 5q deletion suggested by LSI FISH analysis.

Volkert *et al*., suggested that the type of cytogenetic abnormality leading to loss of 5q may harbor important prognostic information. A wide series of MSD and AML adult patients were taken into account in this work. This study showed that the two main commonly deleted regions (CDR) have distinct prognostic value. The CDR1 (5q32) is present in patients with 5q- MDS and isolated 5q deletion, being associated with a good prognosis. The CDR2 (5q31) is present in aggressive MDS and high-risk AML, and has been associated to complex karyotypes and demonstrated a worse prognosis [9].

Interestingly, our patient presented a loss of large portions from the long arm of one of the chromosome 5 that comprised 5q22 to 5qter, including both CDR regions. In this large portion lost in our patient, the literature includes *APC, EGR1, CTNNA1, DIAPH1, NPM1, GLRA1, RPS14, UBE2D2* genes whose disruption of expression may lead to defects in hematopoiesis in mice and other defects in core process to cell development. Lastly, these defects would lead to progression towards AML or 5q- MDS [10].

Harrison *et al*., succeeded in establishing that patients with 5q abnormalities (11/729) has a Hazard Ratio =3.75 (1−14, *p* = 0.01) in relation to patients without these abnormalities, when studying the event free survival [1]. However, it was impossible to find similar statistical results in the other survival outcomes (disease free survival and overall survival), possibly because of the small number of patients in this subgroup. Both COG protocols and United Kingdom Medical Research Council Treatment Trials AML 10 and 12 stratify these patients in the high-risk arm what makes them candidates to allogeneic bone marrow transplantation (BMT).

In another clinical study, Von Neuhoff *et al*., was unable to demonstrate the worst prognostic implication of aberrations in chromosome 5q (14/454) [4]. Although, it is not possible to determine if this inability was also due to a small number of patients. Patients with these types of cytogenetic abnormalities are classified in AML-BFM 2012 protocols as an intermediate risk group, and are not primarily directed to allogeneic BMT. Our patient was treated under the above-mentioned protocol and, because of a late achievement of complete remission; he was posteriorly stratified to high-risk group and submitted to BMT. Thus, if this uncommon breakpoint in 5q22 within complex karyotype has a prognostic impact, more studies with a wide series of AML cases should be performed to confirm our cytogenetic data.

## Conclusion

Here, we described a case presenting an uncommon deletion 5q22 associated with novel abnormalities involving both chromosomes 2, revealed by MCB and BAC probes, as defined: 46,XY,t(2;14)(q23;q32.2),t(2;15)(p23;q15),del(5)(q22-qter) in a child that showed a very poor clinical response to treatment. The knowledge of more complete descriptions of such cases harboring 5q- abnormalities, obtained through the application of molecular cytogenetic approaches, are necessary to precisely stratify the risk of this rare subgroup in childhood AML.

## Consent

Written informed consent was obtained from the patient for publication of this Case report and any accompanying images. A copy of the written consent is available for review by the Editor-in-Chief of this journal.

## Abbreviations

AML: Acute myeloid leukemia
APC: Adenomatous Polyposis coli
BAC: Bacterial artificial chromosome
BMT: Bone marrow transplantation
CD: Cluster differentiation
COG: Children Oncology Group
CTNNA1: Catenin Alpha 1
DIAPH1: Diaphanous–Related Formin 1
EGR1: Early growth response 1
FISH: Fluorescence in situ hybridization
GLRA1: Glycine Receptor Alpha 1
HLA-DR: Human leukocyte antigen-D related
IGH: Immunoglobulin heavy
IREM: Immune receptor expressed by myeloid cells
LSI: Locus specific
MCB: Multicolor chromosome banding
MDS: Myelodisplastic Syndrome
MPO: Mieloperoxidase
NPM1: Nucleophosmin 1
RPS14: Ribosomal Protein S14
TdT: Terminal Deoxynucleotidyl Transferase
UBE2D2: Ubiquitin-Conjugating Enzyme E2D 2
